# Optical Coherence Tomography Angiography Evaluation of Retinal Microvasculature Before and After Carotid Angioplasty and Stenting

**DOI:** 10.1038/s41598-019-51382-8

**Published:** 2019-10-14

**Authors:** Chia-Wei Lee, Hui-Chen Cheng, Feng-Chi Chang, An-Guor Wang

**Affiliations:** 1 Department of Ophthalmology, Fu Jen Catholic University Hospital, Fu Jen Catholic University, New Taipei City, Taiwan; 2 Department of Ophthalmology, Taipei Veterans General Hospital, Taipei, Taiwan; 3 School of Medicine, National Yang-Ming University, Taipei, Taiwan; 4 Program in Molecular Medicine, School of Life Sciences, National Yang-Ming University, Taipei, Taiwan; 5 Department of Life Sciences and Institute of Genome Sciences, School of Life Sciences, National Yang-Ming University, Taipei, Taiwan; 6 Department of Radiology, Taipei Veterans General Hospital, Taipei, Taiwan

**Keywords:** Retinal diseases, Medical research, Vascular diseases

## Abstract

The aim of the study was to evaluate the influence of carotid angioplasty and stenting (CAS) on retinal microvasculature using optical coherence tomography angiography (OCTA) in patients with severe carotid stenosis. 20 patients with severe carotid stenosis underwent comprehensive ophthalmic examinations and OCTA before and one month after CAS. Automated algorithms were used to quantify vessel density in the macular superficial vascular complex (SVC), deep vascular complex (DVC), and radial peripapillary capillary (RPC) around the optic disc. Eyes on the operated side constituted the ipsilateral eye group, and the other eye constituted the fellow eye group. In the ipsilateral eye group, the vessel density in the DVC increased significantly after stent implantation (*P* = 0.010), but the vessel density change in the SVC was not statistically different (*P* = 0.999). In the fellow eye group, the vessel density in the SVC (*P* = 0.028) and DVC (*P* = 0.034) were significantly increased after stent implantation. The vessel density in the RPC did not significantly change in the ipsilateral (*P* = 0.363) or fellow (*P* = 0.878) eye groups. This study shows that unilateral CAS for severe carotid stenosis increases macular vessel densities in both eyes.

## Introduction

Carotid stenosis is one of the major causes of cerebral infarction and contributes to 15–20% of all ischemic strokes^1^. About 5–10% of all individuals aged 70 years or older suffer from carotid stenosis with ≥50% luminal narrowing^2^. Clinical manifestations of carotid stenosis, including acute focal neurologic deficits or chronic ocular ischemia syndrome, reflect the hypoperfusion of the ipsilateral hemisphere or ocular tissue. Several randomized controlled trials established the benefits of carotid revascularization for preventing cerebrovascular events in patients with carotid stenosis^3^. Carotid angioplasty and stenting (CAS) has gained popularity as a revascularization strategy in recent years and is an endovascular procedure utilizing balloon angioplasty and deployment of a stent over the plaque to widen the obstructed artery. With the rapid development of embolic protection devices and advances in techniques, CAS is now a less invasive alternative and has comparable outcomes to traditional carotid endarterectomy (CEA)^4^.

As one of the highest energy-consuming tissues in the body, the retina is particularly vulnerable to ischemia^5^. To meet the metabolic demands of retinal tissue, the arterioles and venules bifurcated from the retinal arteries and veins are organized into several interconnecting retinal vascular plexuses. The retinal vasculature can be viewed directly, offering a readily accessible window to monitor vascular and circulatory function. A growing body of research indicates that abnormal retinal microvascular features may serve as a novel biomarker reflecting the severity of underlying cardiovascular, neurodegenerative, and microvascular disease^6–9^. Since blood flow to the retina is predominantly supplied by the internal carotid artery (ICA), we hypothesized that retinal microvasculature changes after CAS may reflect the disease status or therapeutic effects in patients with severe carotid stenosis.

The emergence of optical coherence tomography angiography (OCTA) revolutionized the way of visualizing and quantifying retinal microvasculature. OCTA is a novel, non-invasive imaging modality that generates vascular flow maps across the retina and choroid, thereby constructing a three-dimensional image of the retinal vasculature. Several studies have shown that OCTA can be a reliable tool for the qualitative and quantitative assessments of the retinal, choroidal, or optic nerve vessels during various ocular pathological or physiological changes^10–12^. Recent studies have also demonstrated the ability of OCTA to quantify retinal microvascular changes in monitoring cardiovascular risk^13,14^. The present study used OCTA to quantify the microcirculation of the retina and optic nerve head. The aim was to investigate the influence of CAS on the retinal and peripapillary vascular plexuses in patients with severe carotid stenosis.

## Methods

### Study design and participants

This retrospective study adhered to the tenets of the Declaration of Helsinki and was approved by the Institutional Review Board of Taipei Veterans General Hospital. A waiver of informed consent was granted by the approving Institutional Review Board. From August 2015 to December 2017, patients were recruited who were diagnosed with severe carotid stenosis and underwent unilateral CAS. The indication for the surgical procedure followed the criteria set by the North American Symptomatic Carotid Endarterectomy Trial (NASCET)^3^. Eyes with ipsilateral carotid stenosis constituted the study group (ipsilateral eye group), while the fellow eyes constituted the other group (fellow eye group). All patients underwent a comprehensive ophthalmologic examination 1–2 days before and 4–5 weeks after CAS, that included measurements of Snellen best-corrected visual acuity (BCVA), slit-lamp biomicroscopy, color fundus photography, and indirect ophthalmoscopy. Standard automated perimetry examinations were conducted using the Swedish Interactive Threshold Algorithm standard 30-2 program (Humphrey Field Analyzer; Carl Zeiss Meditec, Dublin, CA). Reliability criteria were established as less than 20% fixation losses, less than 33% false positive errors, and less than 33% false negative errors. To look for delayed complications, outpatient follow-up appointments were arranged for 1 and 3 months post surgery and patients underwent carotid artery ultrasonography or magnetic resonance angiography 4–5 weeks following CAS. Exclusion criteria included the following: (1) a history of glaucoma, coexisting retinal disease, optic neuropathy, uveitis, ocular trauma, or intraocular surgery (except for uncomplicated cataract surgery), (2) complications associated with CAS within 3 months, and (3) failure to confirm stent patency by carotid artery ultrasonography or magnetic resonance angiography.

### Acquisition of OCT and OCTA images

**Figure 1:**
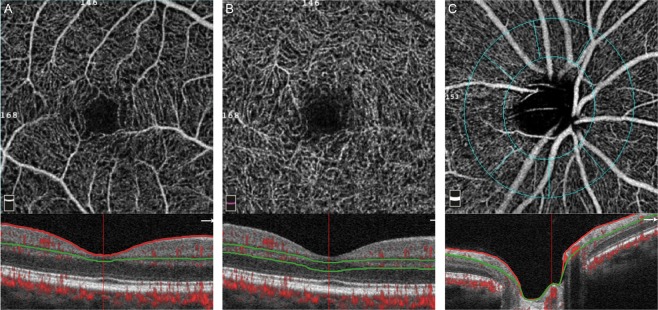
Representative en face scans from optical coherence tomography angiography of the macula and circumpapillary region with corresponding b-scan segmentation. Superficial vascular complex (**A**); deep vascular complex (**B**); and radial peripapillary capillaries (**C**) located in an elliptical ring with a radius of 0.75 mm. Macular vessel density measurements were acquired in a 3 × 3 mm area around the fovea, and the vessel densities in the radial peripapillary capillary plexus layer were acquired in the circumpapillary region of 4.5 × 4.5 mm. The red lines and green lines on the b-scan show the segmentation boundaries for vessel density measurements for each plexus.

The structural OCT scan was performed using a spectral domain Cirrus HD-OCT (software version 6.5.0.772; Carl Zeiss Meditec, Inc., Dublin, CA). The average circumpapillary retinal nerve fiber layer (RNFL) was imaged using the Optic Disc Cube 200 × 200 scan protocol, which evaluated RNFL thickness along a 3.46 mm diameter circle centered on the optic nerve head. The ganglion cell complex (GCC) thickness, which includes the RNFL, ganglion cell layer, and inner plexiform layer (IPL), was measured in an elliptical annulus (vertical radius of 2 mm, horizontal radius of 2.4 mm) in the macula using the Macular Cube 512 × 128 scan protocol. The OCTA imaging and central macular thickness were evaluated with the XR Avanti® AngioVue (software version 2016.2.0.35; Optovue Inc., Fremont, CA). A detailed description of the angioflow vessel density measurement has been described elsewhere^15^. In brief, vessel density was calculated as the percentage area occupied by flowing blood vessels in the segmented region. Patients underwent OCTA scans using a 4.5 × 4.5 mm field of view centered on the optic disc and a 3.0 × 3.0 mm field of view centered on the macula (Fig. 1). Based on the default settings, the whole en face image vessel density of the superficial vascular complex (SVC) was segmented with an inner boundary that was 3 µm posterior to the internal limiting membrane (ILM) and an outer boundary that was 15 µm posterior to the outer aspect of the IPL. The whole en face image vessel density of the deep vascular complex (DVC) was segmented with an inner boundary that was 15 µm posterior to the outer aspect of the IPL and an outer boundary that was 70 µm posterior to the outer aspect of the IPL. The vessel density of the radial peripapillary capillaries (RPC) in the circumpapillary area was calculated in the region defined using a 0.75 mm-wide elliptical annulus delineated by automated fitting extended from the optic disk margin. The segmentation to depict the RPC was between the ILM and the outer limit of the RNFL. Two ophthalmologist (C.W.L. and H.C.C.) reviewed scans and excluded non-qualified images, which were defined as images with (1) a signal strength index <30, (2) automated segmentation errors, (3) local block signal, and (4) motion artifacts or doubling artifacts^16^. The vessel densities from qualified images were included in the pre-post CAS analysis.

### Interventional procedure

The CAS procedure was performed by an experienced interventional neuroradiologist (C.F.C.). Patients were given a dual antiplatelet premedication consisting of orally administered aspirin (300 mg) and clopidogrel (75 mg) at least 3 days before carotid stenting. A transfemoral arterial approach was used to obtain a complete neuroangiogram of the supra-aortic arteries and their branches, including the bilateral carotid, bilateral vertebral, and bilateral subclavian arteries. The degree of stenosis was calculated according to the method used in the NASCET^3^. After intravenous injection of 3000 to 5000 IU heparin, a 6 Fr. guiding sheath was placed in the common carotid artery (CCA) to be stented. Prior to stenting, the distal filter of the cerebral protection device was passed through the stenotic lesions and was opened in a straight segment of the ICA. Balloon predilatation and a self-expanding stent were then deployed. In the postoperative period, dual antiplatelet therapy (75 mg clopidogrel and 100 mg aspirin daily) was administered to the patients for 1 to 3 months, followed by 100 mg aspirin for life.

### Statistical analysis

The statistical analyses were conducted using SPSS (version 22.0; IBM-SPSS Inc., Chicago, IL). The Snellen visual acuity was converted to the logarithm of the minimal angle of resolution (logMAR) for comparison. Baseline structural parameters measured by OCT were analyzed using the non-parametric Wilcoxon signed-rank test to evaluate the difference between groups. The BCVA, mean deviation (MD), and vessel density in the SVC, DVC, and RPC were compared using the Wilcoxon signed-rank test at baseline and after stenting in both groups. All measurements were described as the mean ± standard deviation. All p values were based on two-tailed tests and were considered statistically significant if <0.05.

## Results

A total of 34 patients met the diagnostic criteria of severe carotid stenosis and underwent uncomplicated CAS. Among them, 10 patients with incomplete ophthalmic examinations or OCTA images before and after CAS were excluded. We also excluded three patients with a history of glaucoma and one patient with previous ischemic optic neuropathy. The remaining 20 patients constituted the subjects of this study. The patients’ demographics, presenting symptoms of carotid stenosis, and ocular characteristics of the ipsilateral and fellow eyes are summarized in Table 1. The severity of carotid stenosis was 80.13 ± 11.48% (60–99%). There was no statistically significant difference among the groups in terms of BCVA, average CMT, average RNFL thickness, or average GCC thickness before stenting.

**Table 1:** Baseline Data for Study Participants and Ocular Characteristics of the Ipsilateral and Fellow Eye Groups.

Variables	Subjects (n = 20)		P*
**Demographics**			
Age (year)^†^	64.8 ± 9.1		
Sex (male/female)	18/2		
Laterality, R/L	7/13		
**Systemic condition**, ***n*** **(%)**			
Hypertension	13 (65%)		
Diabetes mellitus	7 (35%)		
Hyperlipidemia	12 (60%)		
CAD	4 (20%)		
Smoking	10 (50%)		
RT for NPC	6 (30%)		
RT for OPC	2 (10%)		
**Symptoms of carotid stenosis**			
Asymptomatic	2 (10%)		
Amaurosis fugax	1 (5%)		
Transient ischemic attack	6 (30%)		
Ischemic stroke	11 (55%)		
**Severity of carotid stenosis (%)** ^**†**^	80.13 ± 11.48		
Ocular characteristics^†^	Ipsilateral eye	Fellow eye	
BCVA (logMAR)	0.09 ± 0.19	0.03 ± 0.12	0.183
CMT (µm)	256.15 ± 16.48	253.25 ± 14.32	0.468
RNFL thickness (µm)	97.85 ± 9.93	97.50 ± 8.06	0.831
GCC thickness (µm)	83.89 ± 6.32	82.00 ± 6.63	0.363

CAD = coronary artery disease; RT = radiation therapy; NPC = nasopharyngeal carcinoma; OPC = oropharyngeal cancer; BCVA = best-corrected visual acuity; MAR = minimum angle of resolution; CMT = central macular thickness; RNFL = retinal nerve fiber layer; GCC = ganglion cell complex.

*P values are based on the Wilcoxon signed rank test.

^†^Values are given as the mean ± standard deviation.

In the ipsilateral eye group, the vessel density in the DVC increased significantly after stent placement (47.52 ± 5.53% vs. 51.45 ± 5.51%, *P* = 0.010); while, that in the SVC did not significantly change (43.47 ± 5.00% vs. 44.09 ± 6.03%, *P* = 0.999). In the fellow eye group, the vessel density significantly increased after stent placement in the SVC (42.58 ± 4.68% vs. 44.79 ± 5.49%, *P* = 0.028) and DVC (47.95 ± 4.87% vs. 50.64 ± 5.72%, *P* = 0.034). The vessel density in the RPC did not differ significantly at the 1-month postoperative follow-up, either in the ipsilateral (55.71 ± 5.80% vs. 57.47 ± 4.69%, *P* = 0.363) or the fellow (57.32 ± 4.03% vs. 57.27 ± 6.07%; *P* = 0.878) eye group (Table 2 and Supplementary Fig. S1).

**Table 2:** Comparison of Best-Corrected Visual Acuity, Visual Field Mean Deviation, and Optical Coherence Tomography Angiography Vessel Densities in the Retinal Vascular Plexuses Before and 1 Month After Carotid Angioplasty and Stenting in the Ipsilateral and Fellow Eye Groups.

Variables	Ipsilateral eye group				Fellow eye group			
	n	Pre-stenting	Post-stenting	P*	n	Pre-stenting	Post-stenting	P*
BCVA (logMAR)	20	0.09 ± 0.19	0.10 ± 0.17	0.201	20	0.03 ± 0.12	0.06 ± 0.17	0.413
MD (dB)	11	−3.99 ± 2.16	−2.62 ± 2.43	0.013^†^	11	−4.88 ± 3.50	−2.50 ± 2.09	0.004†
VD (%)								
SVC	12	43.47 ± 5.00	44.09 ± 6.03	0.999	12	42.58 ± 4.68	44.79 ± 5.49	0.028†
DVC	12	47.52 ± 5.53	51.45 ± 5.51	0.010^†^	12	47.95 ± 4.87	50.64 ± 5.72	0.034†
RPC	14	55.71 ± 5.80	57.47 ± 4.69	0.363	10	57.32 ± 4.03	57.27 ± 6.07	0.878

BCVA = best-corrected visual acuity; MAR = minimum angle of resolution; MD = mean deviation; VD = vessel density; SVC = superficial vascular complex; DVC = deep vascular complex; RPC = radial peripapillary capillaries

All values are presented as the mean ± standard deviation.

*P values are based on the Wilcoxon signed-rank test.

^†^Statistically significant.

Four of 20 patients had new retinal emboli in the ipsilateral eye. All of them had previous radiation therapy to the head and neck region. Another patient with hyperlipidemia developed retinal emboli in both eyes after stenting. None of these five patients reported visual symptoms after CAS. A subgroup analysis excluding subjects with a post-stenting embolism was conducted in the ipsilateral eye group. The changes in vessel density in the SVC (42.70 ± 5.45% vs. 43.64 ± 5.27%, *P* = 0.889) and RPC (54.89 ± 5.97% vs. 56.97 ± 4.08%, *P* = 0.333) were not significant; while, the changes in vessel density in the DVC (46.97 ± 5.29% vs. 50.72 ± 4.08%, *P* = 0.012) remained significant.

The mean BCVA in logMAR remained unchanged in both groups (0.09 ± 0.19 vs. 0.10 ± 0.17, *P* = 0.201 for the ipsilateral eye group; 0.03 ± 0.12 vs. 0.06 ± 0.17, *P* = 0.413 for the fellow eye group). The visual field examinations revealed a small but significant improvement in MD in the ipsilateral (−3.99 ± 2.16 dB vs. −2.62 ± 2.43 dB, *P* = 0.013) and fellow (−4.88 ± 3.50 dB vs. −2.50 ± 2.09 dB, *P* = 0.004) eye groups after stenting (Table 2 and Supplementary Fig. S2).

## Discussion

In the present study, we investigated short-term retinal microvasculature changes in patients with severe carotid stenosis following CAS. In the ipsilateral eye group, the macular SVC vessel density measured by OCTA did not change markedly; while, the DVC vessel density increased 1 month after CAS. In contrast, a statistically significant increase in vessel density was observed in both the SVC and DVC in the fellow eye group. Previous studies demonstrated that unilateral carotid revascularization may improve cerebral blood flow and metabolism not only in the ipsilateral hemisphere but also in the contralateral hemisphere^17–19^. The improvements in bilateral retrobulbar ocular blood flow and electrophysiological responses of the retina were also identified^20,21^. Collateral blood flow supplying the hemodynamically compromised hemisphere came at the expense of reduced global cerebrovascular reserve. Alleviation of the unilateral carotid stenosis may lessen the reliance on collateral circulation from the contralateral hemisphere, result in blood flow redistribution and restore the ocular and retinal perfusion on both sides. In addition, our patients received medication treatments of underlying vascular risks through the study period. How those medications alter systemic microvascular regulation and retinal microvasculature were not studied in detail. Assuming that retinal microvasculature changes in response to systemic microvascular function, increased retinal vessel density in both eyes may attribute to the longitudinal effect of medications. In consistent with our findings, a recent study using OCTA observed a reduced vessel density in patients with CAS when compared with healthy controls and the vessel density increased significantly after CEA in the ipsilateral and in the contralateral eye^22^.

**Figure 2:**
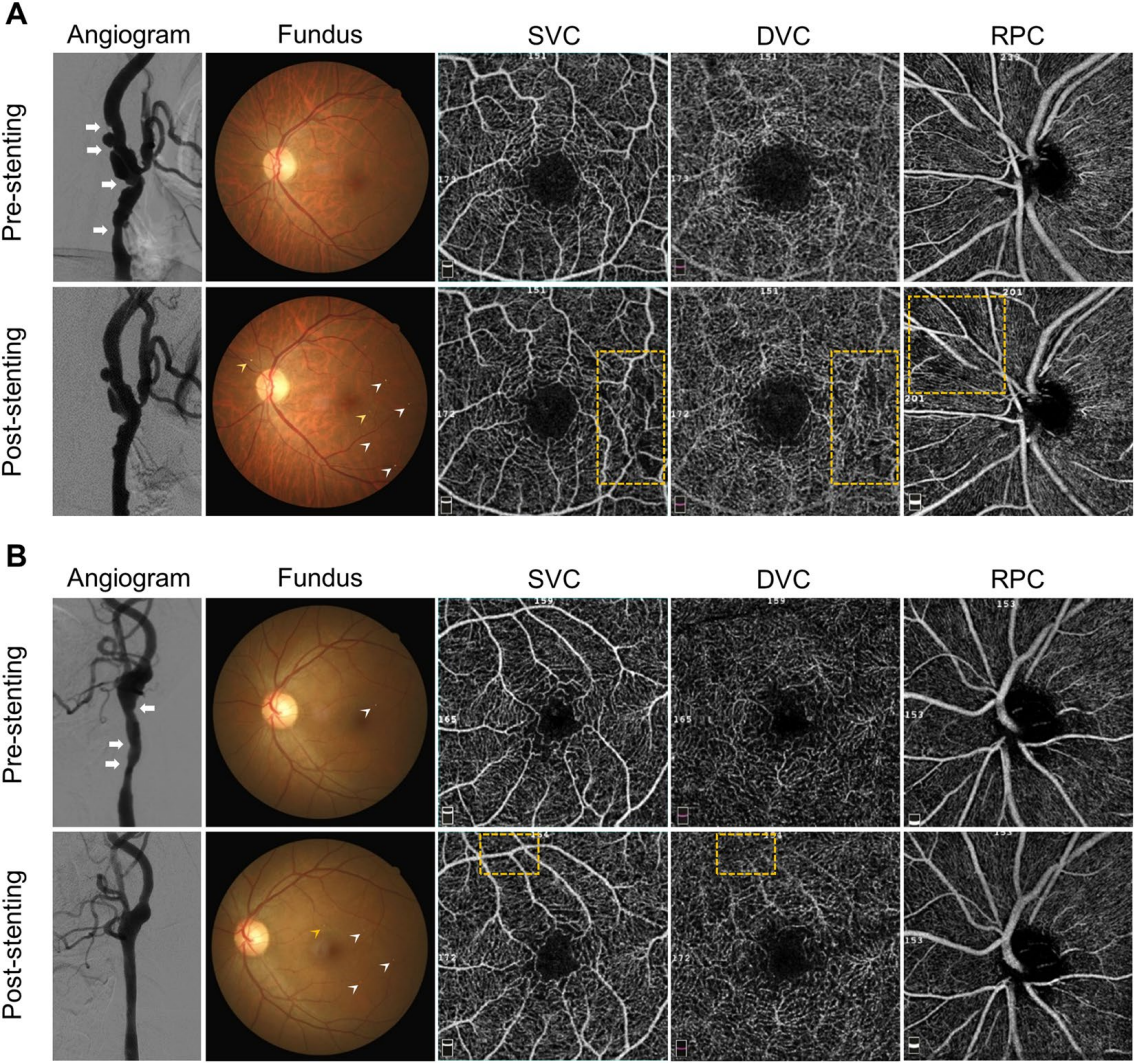
Angiographic characteristics of two cases with radiation-induced carotid stenosis and the effects of retinal emboli on retinal vascular plexus perfusion measured by optical coherence tomography angiography (OCTA). (**A**) Case 1: A 58-year-old man who received radiation therapy for nasopharyngeal carcinoma underwent carotid angioplasty and stenting (CAS). The angiogram showed 80% stenosis of the left internal carotid artery (ICA) with long segmental narrowing and ulcerated plaques in the carotid bifurcation region (*arrows*). Two self-expandable stents were placed from the left ICA to common carotid artery (CCA). At 1-month follow-up, color fundus photography revealed multiple intra-arterial emboli (*arrowheads*) deposited in the arterioles and capillaries temporal to the fovea in the ipsilateral eye. The *yellow arrowhead* indicated retinal emboli deposited within the OCTA scan area. En face OCTA demonstrated an increased area of flow void in the corresponding vascular territories (*yellow* square). (**B**) Case 2: A 45-year-old man underwent CAS for symptomatic carotid stenosis as a late sequelae of radiotherapy. A retinal embolus was detected before stent placement. Long segment stenosis and plaque ulceration were detected involving the left CCA and ICA *(arrows*) and a self-expandable stent was deployed from the left proximal ICA to the CCA. Note that the flow signals at the segmentation of the SVC and DVC were not significantly reduced despite the deposition of one new retinal emboli (*yellow arrowhead*) in the corresponding scan region (*yellow square*). SVC = superficial vascular complex; DVC = deep vascular complex; RPC = radial peripapillary capillaries.

Five patients had clinically silent retinal emboli after CAS, four of whom had been treated with radiation for head and neck cancer. When compared with non-irradiated cases, radiation-induced carotid stenosis were more diffuse and often accompanied by higher incidences of unstable plaques that are prone to rupture (Fig. 2)^23,24^. A much higher rate of new ischemic events during the early post-stenting period has been reported in patients with radiation-induced carotid stenosis^24^. In agreement with published results, our findings suggested that the increased incidence of retinal emboli after CAS might be attributed to plaque vulnerability in patients treated with radiation.

In the ipsilateral eye group, the finding that the vessel density in the SVC remained unchanged did not support our speculation described above. The relatively small sample size may not have been sufficiently powered to detect a difference. In addition, most of the retinal emboli were lodged in retinal arterioles, which are included in the segmentation of the macular SVC in the study design. Intravascular procedures are a well-known precipitating factor for thromboembolic events. Embolization resulting in retinal hypoperfusion following CAS, despite the use of filter protecting devices, has been reported^25^. Hence, ongoing deposition of visible and nonvisible retinal emboli during the postoperative period may lead to focal capillary dropout (Fig. 2A) and the flow signals near the occluded retinal arterioles and capillary networks may not increase or even be reduced, which offsets the benefit of unilateral CAS to ocular circulation. Meanwhile, since most atherosclerotic lesions are located at the proximal ICA and the carotid bifurcation, it is preferable to deploy the stent from the ICA into the CCA. The stent crosses the orifice of the external carotid artery and turbulence might occur when flow passes through the mesh of the stent wall to the external carotid artery. In patients with ophthalmic artery atherosclerosis whose ocular perfusion was maintained via collateral circulation from the branches of the external carotid artery, stent deployment may alleviate the stenosis of the ICA while partially compromising flow in the external carotid artery system^26^. It should also be noted that retinal emboli visible with fundus photography do not always block vascular flow signals in the corresponding position (Fig. 2B). Considering that fundus photography provides only limited two-dimensional information, emboli could obstruct just a portion of the cross-sectional area of the vessel lumen, preserving vascular perfusion^27^.

The RPC comprise a unique plexus of capillary beds within the RNFL around the optic disc. A histological study revealed that the RPC have a distinct morphological appearance: long, straight capillaries running parallel to the nerve fiber bundles with rare anastomoses to other vessels^28,29^. In healthy subjects and glaucoma patients, there was a direct correlation between RPC vessel density and RNFL thickness^30^. According to our study, the vessel density in the RPC did not change significantly in both groups after stenting. It is worth mentioning that by using the automated segmentation algorithm for RPC analysis, both the RPC networks and large peripapillary vessels are in the region of interest. Holló reported a discrepancy where there was significant loss of the peripapillary RNFL thickness and no progression in the RPC vessel density in a glaucoma progression study^31^. He reanalyzed those images using a recently released Angiovue OCT software update (Phase 7) to remove flow signals from large peripapillary vessels and this resulted in the detection of significant vessel density progression in the same hemifield where there was RNFL thickness loss. Similarly, the flow signals from large peripapillary vessels may attenuate the significance of RPC vessel density changes in the current investigation.

Previous studies documented improvements in visual acuity, contrast sensitivity, and the visual field following CEA in the ipsilateral eye^20,32^. Although the influence of a learning effect of the automated perimetry cannot be excluded^33^, visual field improvement in both eyes after CAS is consistent with the previously reported functional vision recovery implicated by electrophysiological responses^21^.

The present study has several limitations. First, the analysis was conducted using a relatively small sample size. Second, alterations in retrobulbar hemodynamic parameters were not evaluated by Doppler ultrasonography. In patients with documented retrobulbar blood flow insufficiency, CAS may have more substantial effects on retinal microcirculation when the autoregulatory response fails to maintain a constant ocular blood supply. Moreover, in the absence of a control group, distinguishing the effect induced by CAS from medications on retinal microvasculature is not feasible. Some limitations of our image analysis methodology include that the automated segmentations in the current OCTA systems have generally adopted the concept proposed by Spaide *et al*.^34^ that the retinal vascular plexus can be grouped into superficial and deep vascular plexuses for image analysis. The SVC is mainly composed of major arterioles, capillary networks, and venules. It supplies all other vascular plexuses through vertical pre-capillary arterial segments. When using automated preset boundaries, the DVC consists of two deeper capillary networks above and below the inner nuclear layer. According to the anatomical studies, these capillary networks are referred to as the intermediate and deep capillary plexuses, respectively^35,36^. A recently developed image process, termed the project-resolved algorithm, improves depth resolution and allows visualization of these distinct capillary plexuses in their true anatomical location^37^. Hence, by separating the flow signals in the three capillary plexuses and noncapillary vessels, OCTA with this new algorithm may identify even more pathophysiological changes in the retinal microvasculature. Finally, the scan area was not adjusted by axial length. Ocular magnification effects due to axial length variation produce significant image size changes in extreme short or long eyes^38^. Caution should be taken in making comparisons of scan area across studies.

In conclusion, we found a significant increase in vessel density in the DVC in both the ipsilateral and fellow eyes following CAS. New retinal emboli in the early postoperative period may compromise inner retinal microcirculation in the ipsilateral eye. In patients with severe carotid stenosis, unilateral CAS effectively improved the retinal microcirculation in both eyes. OCTA provides a noninvasive assessment of retinal microvasculature and, along with Doppler ultrasonography, may help establish a new biomarker for predicting and monitoring hemodynamic changes in patients undergoing CAS.

## Supplementary information

Supplementary figures

## Data Availability

The datasets generated and analysed during the current study are available from the corresponding author on reasonable request.
