# Supplementary material for: Optical Coherence Tomography Angiography Evaluation of Retinal Microvasculature Before and After Carotid Angioplasty and Stenting
Source: Sci Rep. 2019 Oct 14;9:14755. doi: 10.1038/s41598-019-51382-8 (PMC6791857; doi:10.1038/s41598-019-51382-8)
Supplement: Supplementary file 1 — Supplementary figures [file 41598_2019_51382_MOESM1_ESM.pdf]

Supplementary information

Optical Coherence Tomography Angiography Evaluation of Retinal Microvasculature Before and After Carotid Angioplasty and Stenting.

Chia-Wei Lee[1], Hui-Chen Cheng[2,3,4,5], Feng-Chi Chang[3,6], An-Guor Wang[2,3,*]

[1]Department of Ophthalmology, Fu Jen Catholic University Hospital, Fu Jen Catholic University, New Taipei City, Taiwan.

[2]Department of Ophthalmology, Taipei Veterans General Hospital, Taipei, Taiwan.

[3]School of Medicine, National Yang-Ming University, Taipei, Taiwan.

[4]Program in Molecular Medicine, School of Life Sciences, National Yang-Ming University, Taipei, Taiwan.

[5]Department of Life Sciences and Institute of Genome Sciences, School of Life Sciences, National Yang-Ming University Taipei, Taiwan.

[6]Department of Radiology, Taipei Veterans General Hospital, Taipei, Taiwan.

* agwang@vghtpe.gov.tw

List of Supplementary materials:

Supplementary Figures: Figure S1, Figure S2

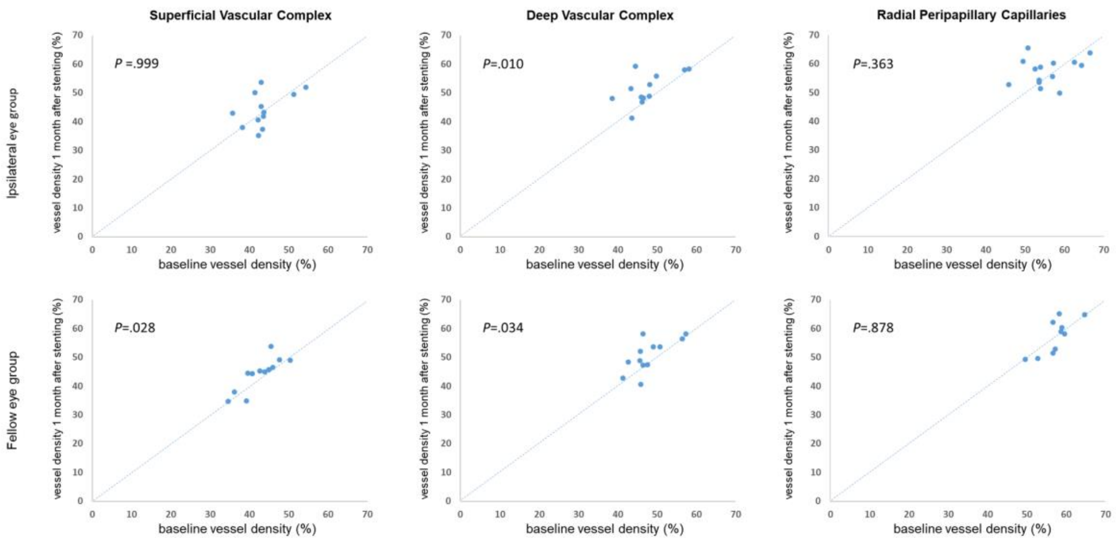

Figure S1. **Scatterplots of baseline vessel density versus post-stenting vessel density in the superficial vascular complex (left column), deep vascular complex (middle column), and radial peripapillary capillaries (right column).**

Points above the line indicate eyes with increased vessel density. **Top row**: ipsilateral eye group; **Bottom row**: fellow eye group.

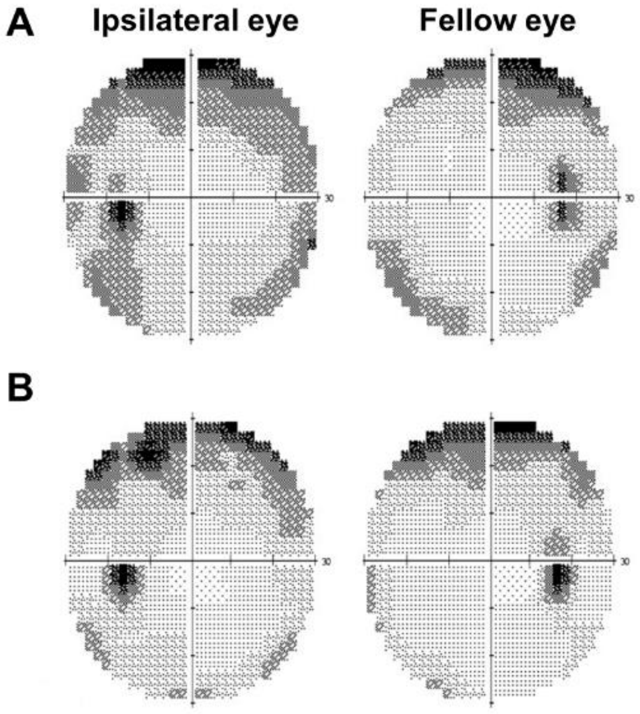

Figure S2. **Visual field improved after carotid artery stenting.**

The Humphrey central 30-2 visual field in a 74-year-old woman with severe stenosis of the left internal carotid artery showed (A) peripheral constriction in both eyes before carotid artery stenting and (B) mild visual field improvement in the ipsilateral and fellow eyes 1 month after stenting.
